# Balloons and Stents in the Endovascular Treatment of Cerebral Aneurysms: Vascular Anatomy Remodeled

**DOI:** 10.3389/fneur.2014.00041

**Published:** 2014-04-08

**Authors:** Michel Piotin, Raphaël Blanc

**Affiliations:** ^1^Department of Interventional Neuroradiology, Foundation Rothschild Hospital, Paris, France

**Keywords:** aneurysm, coiling, balloon remodeling, stent-assisted coil embolization, complications, strategies, angiography, vascular diseases

## Abstract

Wide-neck intracranial aneurysms were originally thought to be either untreatable or very challenging to treat by endovascular means because of the risk of coil protrusion into the parent vessel. The introduction of the balloon remodeling technique (BRT) and later stents specifically designed for intracranial use has progressively allowed these lesions to be endovascularly treated. BRT and stent-assisted coiling technique (SACT) were first designed to treat sidewall aneurysms but, with gained experience and further technical refinement, bifurcation complex-shaped wide-neck aneurysms have been treated by coiling enhanced by BRT and SACT. In this article, we will review and describe the inherent benefits and drawbacks of BRT as well as SACT.

## Introduction

Wide-neck (when the neck is ≥4 mm, or when the dome/neck ratio <1.5–2) intracranial aneurysms were originally thought to be either untreatable or very challenging to treat by endovascular means because of the risk of coil protrusion into the parent vessel. The introduction of the balloon remodeling technique (BRT) and later stents specifically designed for intracranial use has progressively allowed these lesions to be endovascularly treated. BRT and stent-assisted coiling technique (SACT) were first designed to treat sidewall aneurysms but, with gained experience and further technical refinement, bifurcation complex-shaped wide-neck aneurysms have been treated by coiling enhanced by BRT and SACT. In this article, we will review and describe the inherent benefits and drawbacks of BRT as well as SACT. The specific role of flow diverter stents in the endovascular treatment of cerebral aneurysms will not be addressed in this article.

## Balloon Remodeling Technique

### Various techniques of balloon remodeling

The standard coil embolization (stand-alone coiling) technique is limited by its inability to occlude wide-neck aneurysms. The BRT consists in the temporary inflation of a non-detachable balloon across the aneurysm neck during each coil placement to avoid inadvertent coil protrusion into the parent artery as initially described by Moret et al. nearly two decades ago (1). At the end of the procedure, the balloon is removed and no device is left in place in the parent vessel (unless stent placement is subsequently performed). Some balloon catheters allow the placement of a stent at the end of the procedure by inserting the stent into the lumen of the balloon microcatheter after withdrawal of the wire (2). The “classic” BRT, using a single low-compliance balloon, was initially limited to sidewall aneurysms, and was often inadequate for protection of both the neck and arterial branches of complex bifurcation aneurysms. Nowadays, the most popular remodeling balloon microcatheters are the HyperGlide™ (compatible with 0.010″ microguidewire), the Transform™, and the Septer™ (both compatible with 0.014″ microguidewires). Balloons compatible with 0.014″ microwire seem more stable than balloon operating on 0.010″ platforms but induce more deformation of the cerebral arteries during navigation. The Septer™ has two independent lumens, giving to the operator the opportunity to navigate coils or some microstents while the balloon is still inflated. For bifurcated lesions, the use of more compliant balloon (compliance is a mechanical property defined by the propensity of the balloon to change its cylindrical shape to the anatomy of the vessel in which it is inflated) allows the treatment of complex, wide-neck bifurcation aneurysms for which the standard embolization technique would not have permitted safe (regarding the patency of bifurcation arterial branches) endovascular occlusion. In these situations, it is necessary to completely protect the neck to avoid coil protrusion. Several options are available. First, a more compliant balloon can be used to mold the neck and the origin of bifurcation branches (3). The most popular compliant balloons microcatheters are the HyperForm™ (compatible with 0″ microguidewire), the Transform™ C and SC (C and SC for compliant and super compliant, respectively) and the Septer C and SC™ (both compatible with 0.014 microguidewires). An alternative to the use of a super compliant balloon consists in the placement of two balloons instead of one (one balloon in each of the bifurcated arterial branches) (4). The third option consists in the navigation of the balloon through the circle of Willis to cross and protect the aneurysm neck (e.g., to navigate from the internal carotid, the posterior communicating arteries and the P1 segment of both posterior cerebral arteries to protect the neck of a basilar tip aneurysm) (5). Another technique consists in the navigation of a dual-lumen balloon in front of the neck to allow coil deposition through the second lumen of the balloon microcatheter (6). Nowadays, BRT can be used in all aneurysm locations.

### Complications and clinical outcome of BRT

The two most frequent and feared complications of the endovascular treatment of intracranial aneurysms are thromboembolic events and aneurysm perforation. The use of an adjunctive balloon for aneurysm coiling has raised some concerns about potential added morbidity over the standard coiling procedure. In a recent large prospective multicenter study, a consecutive series of patients with ruptured aneurysms (the CLARITY study) who underwent endovascular treatment with either conventional coil embolization or BRT showed that both techniques had similar safety in terms of perioperative complications and clinical outcome (7). The overall rate of treatment-related complications, with or without clinical manifestations, was 17.4% with coil embolization and 16.9% with BRT. The difference in the rates of thromboembolic events, intraoperative rupture, and early rebleeding between the two treatment groups was not statistically significant. The cumulative morbidity and mortality rate related to the treatment in the remodeling group (3.8%) was similar to that in the stand-alone coil embolization group (5.1%). Likewise, the global cumulative morbidity and mortality rates related to both the treatment and the initial hemorrhage did not differ significantly between groups (16.2% with BRT and 19.6% with coil embolization). In the ATENA study (unruptured aneurysms) (8), the overall complication rate, regardless of whether the adverse events led to clinical consequences, was 10.8% for standard coiling of unruptured aneurysms and 11.7% for BRT of unruptured aneurysms. The morbidity and mortality rates did not differ significantly between groups: 3.1% in the standard treatment group and 3.7% in the BRT group, respectively (8). In the Shapiro et al. review article (9), in ruptured aneurysms, the clinical outcome was a symptomatic event or death in 2.7% in the stand-alone coiling group and 1.7% in the BRT group. In unruptured aneurysms, clinical outcome was a symptomatic event or death in 0.6% in the stand-alone coiling group and 0.9% in the BRT group. Table 1 provides with an overview of the rates of complications with BAT.

**Table 1:** **Balloon. remodeling technique and complications with clinical significance**.

	Morbi-mortality	
	Stand-alone coiling (%)	BRT (%)
CLARITY, ruptured aneurysms (7)	5.1	3.8
ATENA, unruptured aneurysms (8)	3.1	3.7
Shapiro et al. review, ruptured aneurysms (9)	2.7	1.7
Shapiro et al. review, unruptured aneurysms (9)	0.6	0.9

### Aneurysm perforation

In the Shapiro et al. review article (9), the rate of intraoperative rupture was 3.4% in ruptured aneurysms treated with standard coiling, 1.7% in ruptured aneurysms treated with the remodeling technique, 1.4% in unruptured aneurysms treated with standard coiling, and 1.8% in unruptured aneurysms treated with the remodeling technique. In the ATENA study (unruptured aneurysms) (8), the rate of intraoperative rupture was 3.2% in the remodeling group and 2.2% in the coiling group. In the Sluzewski et al. personal series (10), the rate of intraoperative rupture was higher in the remodeling (4.0%) compared with the coiling group (0.8%). In the CLARITY study (ruptured aneurysms), the rates of intraoperative rupture were similar in both BRT and stand-alone coiling groups (7).

### Thromboembolic complications

In the Shapiro et al. review (9), the rate of thromboembolic events was similar in patients treated with coiling (8.1%) and remodeling (8.0%). Symptomatic thromboembolic events were encountered in 4.6% of patients treated with coiling and 4.4% of patients treated with remodeling. Death related to thromboembolic events was reported as 1.2% for patients treated with coiling and 0.4% for patients treated with remodeling. In the Layton et al. series (11), the rate of thrombus formation was not significantly different in patients treated with standard coiling compared with the remodeling technique (9 and 14%, respectively). Symptomatic thromboembolic events were also observed in a similar percentage of cases (5% in standard coiling and 7% in remodeling). Similarly, Brooks et al. reported that diffusion-weighted-imaging abnormalities were detected in 32% in the coiling group and 24% in the BRT (12). Conversely, Sluzewski et al. reported that the rate of thromboembolic events was higher in the remodeling group (9.8%) compared with the coiling group (2.2%) (10). In the ATENA study, thromboembolic events occurred in 6.2% in the stand-alone coiling group versus 5.4% in the BRT group (8).

### Role of balloon inflation time for BRT regarding ischemic complications

Critical questions regarding the maximum permissible balloon occlusion time, the minimum effective reperfusion time between inflations, and whether total balloon inflation time or the number of inflations is a higher risk factor of BRT than stand-alone coiling for ischemic complications has been assessed using diffusion-weighted MR imaging (13, 14). For Albayram et al., the only variables found to influence this risk during or after BRT coil placement were microcatheter repositioning, coil removal and repositioning, and size of the aneurysmal neck (13). More recently, Spiotta et al. found that asymptomatic ischemic event rate in this population for BRT embolization was 24.7%, a rate equal to stand-alone coiling of patients treated in the same time period without BRT (14). Both silent and symptomatic ischemic rates were similar in the internal control group. It is possible that the higher rate of antiplatelet therapy in the BRT group is masking a higher ischemic rate. The baseline patient risk factors for ischemic complications identified included older age and diabetes. One possible explanation for this finding is that all patients have intraprocedural showering of emboli, but older and diabetic patients are more likely to have irreversible ischemia attributable to preexisting microvascular disease. Embolic infarcts were more common than watershed infarcts. The total number of inflations times, the maximum occlusion time, minimum reperfusion time between two consecutive inflations, and mean reperfusion time did not appear to be risk factors for thromboemboli. However, higher maximum inflation time was significantly correlated to watershed pattern infarcts.

### Anatomic results of the BRT

The Shapiro et al. literature review does not confirm the Sluzewski et al. findings (9, 10). Both initial and follow-up aneurysm occlusion rates were higher in BRT cases. The initial total occlusion rate was 73% in patients in the BRT group and 49% of patients in the standard coiling group, subtotal occlusion in 22% in the BRT group and 39% in the coiling group, and incomplete occlusion in 5% in the BRT group and 13% in the coiling group. At follow-up, there were similar results: total occlusion in 72% of patients in the BRT group and 54% of patients in the standard coiling group, subtotal occlusion in 17% in the BRT group and 34% in the coiling group, and incomplete occlusion in 10% of the BRT group and 11% of the coiling group. According to the ATENA and CLARITY studies, results are possibly different in unruptured and ruptured aneurysms. In ATENA (unruptured aneurysms), immediate anatomic results reported were similar in both stand-alone coiling and BRT groups (complete occlusion in 59.8% of aneurysms in the stand-alone coiling group and 59.8% of aneurysms in the BRT group) (8). In CLARITY (ruptured aneurysms) (7), immediate anatomic results were different, the rate of adequate angiographic aneurysm occlusion being significantly higher in the BRT group (94.9%) than in the stand-alone coil embolization group (88.7%). A recent meta-analysis from Shapiro et al. demonstrated that although balloon use was associated with superior initial and follow-up angiographic occlusion rates (9).

## Stent-Assisted Coiling of Intracranial Aneurysms

### Rationale for the stenting of intracranial aneurysms

The widespread acceptance of coiling has been hindered by the potential for aneurysm to recur over time after coiling (15). This issue is even more relevant for large aneurysms for which angiographic recurrence is more likely than smaller lesions (16). However, fusiform and some wide-neck aneurysms remained unaddressed by both reconstructive surgical and endovascular techniques until the introduction of dedicated intracranial self-expandable stent. Stent deployment across the aneurysm neck, followed by coil packing of the aneurysm, has progressively been more widely adopted, particularly for wide-neck complex aneurysms, in order to stabilize the coil mass inside the aneurysmal sac and to avoid coil herniation into the parent artery (17, 18). Some authors have also advocated using the stent (or several stents deposited in a telescopic fashion to augment mesh density) as a stand-alone procedure to treat fusiform aneurysms, obtaining progressive aneurysm thrombosis without the adjunct of coils within the aneurysm sac (19–23). With gained experience, SACT has been employed to treat a larger range of aneurysms (not only wide-neck and complex aneurysms) with the idea of the likelihood of diminished risk of aneurysm recurrence (24–26). The major current concern is the small size of the parent vessel relative to the diameter of the smallest available stent with inherent potential suboptimal stent deployment. Nevertheless, some stents can be adequately deployed even in vessel smaller than 2 mm (27). Conversely, the use of SACT has brought with it other important considerations, including the necessity of antiplatelet therapy that carries inherent risks of intracranial bleeding (28, 29). Moreover, antiplatelet therapy is limited in the setting of subarachnoid hemorrhage for the majority of the operators (30). The other drawback, even if limited, of SACT is the potential for delayed stent-related issues such as the development of in-stent stenosis and parent vessel occlusion (31–33).

### Close- and open-cell designs for self-expandable stent

There are two major different, close- and open-cell designs for the construction of self-expanding stents dedicated to the intracranial use. The close-cell design makes the stent to work as a whole body (e.g., Enterprise™); thus, a force used at one end will be transmitted to the other end immediately. For a stent with open-cell design (e.g., Neuroform™), each independent segment can serve as a separate fixing device, to enhance apposition of the stent to the arterial wall, and a force used at one end will not be transmitted to the other end so easily. Open-cell design stents better cover the aneurysm neck when compared to close-cell stents, and induce less straightening of the vessel. The open-cell stents have, however, less struts apposing well to the vessel wall compared to close-cell stents (34). Open-cell stents conforms better to vascular tortuosities. However, open-cell stents may show increased opening of cells and outward prolapse of struts into an aneurysm neck when situated at the convexity of the curvature, whereas at the concavity, struts, or stent segments may protrude inward.

When a closed-cell stent is bent, it has less flexibility to conform to a curved or irregular anatomy. The close-cell unsegmented design does not allow the stent to lengthen at the outer curve or to shorten at the inner curve. This limitation in adapting to a vessel curvature will cause flattening of the stent or kinking (35) resulting in incomplete stent apposition. Incomplete stent apposition has recently been found to be a critical factor associated with higher thromboembolic complication rates in SACT embolization of intracranial aneurysms (36). The major advantage of a close-cell stent is ability to be deployed in the vessel lumen and resheated in its delivery microcatheter, allowing the operator to optimize the position of the stent regarding the aneurysm neck. Conversely, an open-cell stent, once partially delivered, cannot be resheated and repositioned owing to its design consisting in independent stent segments soldered by connectors.

There are two different types of close-cell stents: laser-cut (as the Enterprise™) or woven (as the LVIS™ and the LEO™). Nowadays, the last two stents offer the lowest profile to be delivered in a 0.017″ inner lumen microcatheter.

### Stenting techniques

**Figure 1:**
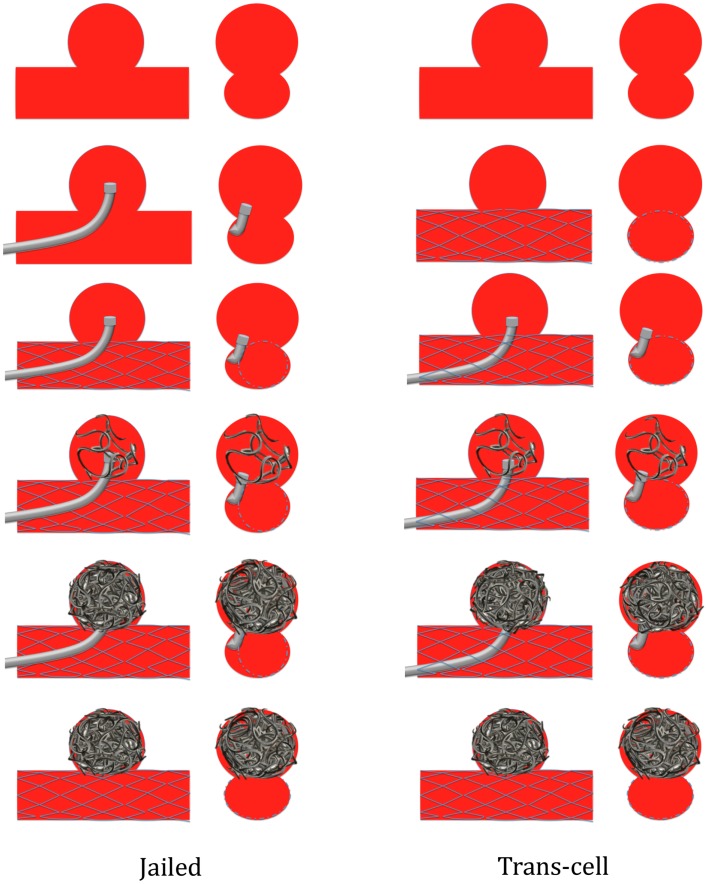
**Jailed-catheter and trans-cell techniques**.

Four options may be proposed. Firstly, the coil delivery microcatheter can be placed first within the aneurysm lumen to allow coil delivery and then the stent is positioned and immediately delivered across the aneurysm neck (jailed-catheter technique). Secondly, the stent can be first delivered across the aneurysm neck and then the coiling microcatheter is placed within the sac through the stent struts (trans-cell technique) (Figure 1) (37). Finally, the aneurysm can be coiled with or without the balloon remodeling technique and then the stent is delivered across the aneurysm neck at the end of the procedure, aiming at decreasing the recanalization rate by diminishing intra-aneurismal flow by diversion and also by creating a mesh at the level of the neck to be colonized and covered by endothelial cells (25). On the other hand, delivering the stent prior to aneurysm coiling has some drawbacks. Firstly, the jailed-catheter technique does not offer the possibility to modify the microcatheter position within the aneurysm sac, resulting in many instances in the diminution of aneurysm packing with coils. Secondly, when the stent is deployed prior to the coil delivery microcatheter placement, the operator should be very cautious while catheterizing the aneurysm through the stent strut in keeping with the potential hazard of stent displacement and stent cell impingement (38). A fourth SACT, the stent-jack technique, has been more recently described. It consists in positioning the coil delivery microcatheter first into the aneurysm sac, then navigating a self-expandable stent into the parent vessel without delivering the stent before the first coil is deposited in the sac (39). The first coil is placed into the sac (no matter if a coil loop was slightly protruding into the arterial lumen) with coil deployment aiming at forming the most homogenous framing of the aneurysm sac. As a next step, before coil detachment, the stent is carefully deployed across the neck. Once the stent is delivered, the first coil is detached. If necessary, additional coils are introduced into the aneurysm to obtain circulatory exclusion of the lesion.

### Stenting of bifurcation aneurysms

Stents have been designed originally to treat sidewall aneurysms. Single-stent SACT is suitable for many bifurcation aneurysms, as a stand-alone technique or in conjunction with BRT (the balloon to be placed in one of the bifurcated branch, the stent being delivered in the other branch). More recently, double stenting in a Y or X configuration may be used to treat a subset of wide-necked aneurysms not amenable to reconstruction with a single-stent due to anatomical conformation (40, 41). Y- and X-stent reconstructions enable the endovascular management of otherwise complex, wide-neck cerebral aneurysms and can be performed as safely as single-stent technique in experienced hands with satisfactory results.

#### Y-stent and waffle-cone technique

The Y-stent technique has been developed first to treat wide-neck basilar tip aneurysms (42). It includes the crossing-Y and kissing-Y techniques (43). The crossing Y-stent technique is based on the strategy that a second stent is advanced through the first stent interstices and into the contralateral branch vessel. By contrast the kissing technique, two stents are deployed in a parallel fashion from both daughter arterial branches down to the main arterial trunk, forming a kissing-Y configuration. The first bifurcation vessel to be stented is determined according to the angle between the proximal parent vessel and the arterial branches just distal to the aneurysm; the branch with a sharper angle to be stented before the one with wider angle. In 2004, Horowitz et al. described a single-stent technique to treat broad-neck bifurcation aneurysm consisting in a single-stent to be placed partially into the aneurysm and into the afferent artery, the portion of the stent protruding into the aneurysm fundus providing neck support for the subsequent successful coiling (44).

#### X-stent technique

Anterior communicating artery (AcoA) aneurysms may present with complex anatomic features, often associated with a wide-neck and variety of anomalies. X-configured stent-assisted coiling for treatment of wide-neck and complex AcoA aneurysms, for which otherwise there would be no endovascular treatment alternative (40, 45). Of course, X-stent placement is to be reserved for patients having good-sized A1 segments, bilaterally. The side of the first stent is determined according to the angle between the A1–AcoA complex and the contralateral A2; the A2 with a sharper angle to be stented before the one with wider angle. On the basis of this decision, the first stent is placed across the aneurysm neck, extending from the contralateral A2 to the ipsilateral A1 segment, crossing through the AcoA. Then after, the second stent is crossed from the other side. Both strut crossing and kissing stenting technique have been reported (40, 45–47).

### Effect of SACT on immediate angiographic outcome and at follow-up

Immediate angiographic complete occlusions are obtained less frequently in stented than in the not stented aneurysms. This is because larger aneurysms are more likely to be stented than small aneurysms, and that dual antiplatelet therapy impacts on the immediate intra-aneurysmal thrombosis. Moreover, the use of dual antiplatelet therapy during the procedure in addition to heparin does not favor immediate per procedural sac thrombosis (25). Tight coiling is more difficult to obtain when the stent is implanted prior to coiling, giving less maneuverability to the coiling microcatheter thus resulting in looser aneurysm packing.

Conversely, at follow-up, complete occlusions increased to 73.4% in the stent-assisted group, while it diminished to 54.0% in the no-stent group. For stent-assisted coiling, numerous articles have reported a broad range (13.2–94.4%) of immediate complete occlusion (48–54). However, most mid-to-long-term follow-up series have reported augmented rates of angiographic complete occlusion at follow-up (range 54–81%) (24–26, 49–62). An absence of stent has been identified as one of the most relevant factors for angiographic recurrence (25). This durability can be explained by the combination of biological, geometrical, and hemodynamic mechanisms (63–68). Hemodynamic effects of the stents in the endovascular treatment of aneurysms include disruption of intra-aneurysmal flow pattern, resulting in turbulence, and production of blood stasis within the aneurysm, resulting in aneurysmal thrombosis. This hemodynamic effect seems even more preeminent in case of Y-stenting (65).

### Complications of SACT

There are more procedure-related complications than in the stand-alone coiling. The main cause of morbidity and mortality is thromboembolism. The necessity of dual antiplatelet therapy in SACT is also known to increase the risk of hemorrhagic complications (69). Thromboembolic complications are also more frequent in the stented patients (70). Antiplatelet activity assessment prior to stent delivery allows diminishing the occurrence of such complications by identifying the patients not responding to antiplatelet drugs (71, 72). In a recent review article, Shapiro et al. reported an overall complication incidence of 19%, with an overall death incidence of 2.1%. Thromboembolic issues were most prevalent at close to 10%, leading to death in 0.6% of overall cases. Hemorrhagic complications occurred in 2.2% of cases but carried a higher association with mortality, accounting for 0.9% of overall deaths. Coil-related technical issues were infrequent (2%) and almost always asymptomatic. Complication rates decrease overtime while the operator practice of stenting increased showing also the effect of a learning curve (26). More recently, Nishido et al. have reported 7.0% of ischemic and 2.3% of hemorrhagic complications with an overall rate of procedure-induced mortality of 2.7% with SACT (73). Geyik et al., in a series of 500 consecutive SACT aneurysms, reported 5.6% of thromboembolic and 0.8% of hemorrhagic complications, with a procedure-related mortality of 0.8% (74). Table 2 provides with an overview of the rates of complications with SACT.

**Table 2:** **Stent-assisted coiling technique complications with clinical significance**.

	Morbi-mortality	
	Stand-alone coiling and BRT	SACT
Nishido et al. (73) unruptured and ruptured aneurysms	5.6%	9.4%
Shapiro et al. (10) review, unruptured and ruptured aneurysms	NA	12.2%

### SACT in the acute setting of SAH

The use of SACT in the acute setting of subarachnoid hemorrhage remains controversial despite positive results reported in limited series (75, 76). It appears that the risks of intracranial hemorrhage are augmented in this particular condition. In their review article, Bodily et al. reported that SACT in ruptured aneurysms could be performed with high degrees of technical success, but adverse events appeared more common and clinical outcomes were likely worse than those achieved without stent assistance (30). The optimal antiplatelet medication during acute-phase treatment has yet to be determined, and a longer follow-up series is needed to evaluate the long-term efficacy and safety of stent-assisted coil embolization during acute SAH. In a series of 36 patients, Golshani et al. found that SACT was an option for treatment of ruptured wide-neck ruptured aneurysms and for salvage treatment during unassisted embolization of ruptured aneurysms but complication rates appeared to be higher than for routine clipping or coiling of cerebral aneurysms (28). This applies even more dramatically to the patients requiring ventriculostomy (77).

### When to use BAT

In our center, BAT is used in slightly over 60% of the cases for both ruptured and unruptured aneurysms. BAT provides the ability to avoid coil protrusion and to protect from the deleterious effect of a massive subarachnoid hemorrhage in case of aneurysm perforation. The balloon can be immediately inflated in case of dome (re)rupture during intervention. The main drawback of the technique is the need for dual femoral approach, augmenting the potential for access site complications. Alternatively some operators advocate the use of a single guiding catheter (with a minimal inner lumen diameter of 0.070″), allowing the navigation of both the balloon and the coil delivery microcatheters. The main drawback of this single guiding catheter technique is the potential inadvertent forward and backward movements of one of the two microcatheters while manipulating the second one.

### When to use SACT

The need for dual antiplatelet therapy makes the SACT to be avoided in acutely ruptured aneurysms. In this setting, SACT should be reserved as a bail-out procedure to avoid parent vessel closure when inadvertent coil protrusion is threatening. We rather prefer to perform a partial coiling to protect the fundus of the aneurysm to avoid early rebleed and to carry back the patient to the angiographic suite a few weeks later to optimize the aneurysm occlusion with the adjunct of a stent if required.

### Antiplatelet therapy management for elective SACT

**Figure 2:**
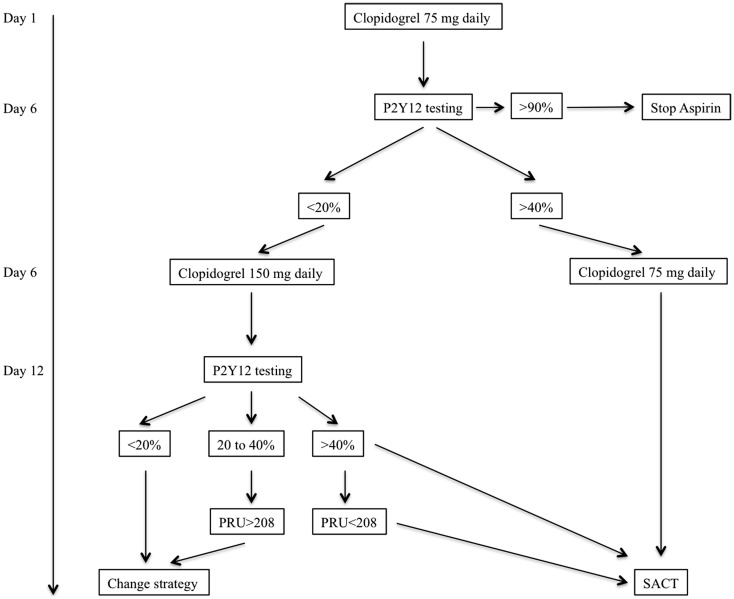
**Flow chart of our antiplatelet therapy assessment prior to elective stent-assisted coiling procedures**. The patient is given clopidogrel and aspirin (250 mg daily) at Day 1. Because clopidogrel is a prodrug treatment should be initiated 5–6 days before efficacy assessment. PRU, platelet reaction unit; SACT, stent-assisted coiling technique.

Our standard dual antiplatelet regimen is based on the oral administration of clopidogrel and aspirin. Instead of using a loading dose of clopidogrel we initiate dual antiplatelet therapy (clopidogrel and oral aspirin) 10–15 days prior to the procedure. For antiplatelet activity assessment we use the VerifyNow (Accumetrics, San Diego, CA, USA) system which a bedside system that is requiring minimal manipulation. This measurement is based on the principles of optical aggregometry. Because low responder patients may not be recognized with loading dose we rather prefer to initiate the treatment with a standard dose of Clopidogrel (75–150 mg daily). Our protocol has been standardized and applied to all patients for whom elective aneurysm treatment with SACT is scheduled (Figure 2).

## Conclusion

Despite the fact that aneurysms treated by the remodeling technique are different from aneurysms treated with standard coiling, the safety of both techniques is similar with a higher anatomic efficacy of the remodeling technique. Accordingly, wide use of the remodeling technique can be proposed. SACT is associated with a higher mortality compared with coiling with or without remodeling and remains more hazardous than stand-alone or BRT coiling in keeping with augmented risks of both ischemic and hemorrhagic insults. However, SACT reduce significantly angiographic recurrence, a factor that alters the results of endovascular treatment over surgical clipping. In the setting of subarachnoid hemorrhage, SACT should be reserved to the otherwise untreatable aneurysm, even if BRT is used, in order to protect the patient from early rebleeding. The optimal antiplatelet regimen in the acute setting has not been determined yet.

## Conflict of Interest Statement

The authors declare that the research was conducted in the absence of any commercial or financial relationships that could be construed as a potential conflict of interest.
